# Application of Metallic Nanoparticles and Their Hybrids as Innovative Sorbents for Separation and Pre-concentration of Trace Elements by Dispersive Micro-Solid Phase Extraction: A Minireview

**DOI:** 10.3389/fchem.2021.672755

**Published:** 2021-05-04

**Authors:** Ingrid Hagarová, Lucia Nemček

**Affiliations:** Institute of Laboratory Research on Geomaterials, Faculty of Natural Sciences, Comenius University in Bratislava, Bratislava, Slovakia

**Keywords:** metallic nanoparticles, carbon-based nanomaterials, dispersive micro-solid phase extraction, aqueous samples, ultratrace elements

## Abstract

It is indisputable that separation techniques have found their rightful place in current analytical chemistry, considering the growing complexity of analyzed samples and (ultra)trace concentration levels of many studied analytes. Among separation techniques, extraction is one of the most popular ones due to its efficiency, simplicity, low cost and short processing times. Nonetheless, research interests are directed toward the enhancement of performance of these procedures in terms of selectivity. Dispersive solid phase extraction (DSPE) represents a novel alternative to conventional solid phase extraction (SPE) which not only delivers environment-friendly extraction with less solvent consumption, but also significantly improves analytical figures of merit. A miniaturized modification of DSPE, known as dispersive micro-solid phase extraction (DMSPE), is one of the most recent trends and can be applied for the extraction of wide variety of analytes from various liquid matrices. While DSPE procedures generally use sorbents of different origin and sizes, in DMSPE predominantly nanostructured materials are required. The aim of this paper is to provide an overview of recently published original papers on DMSPE procedures in which metallic nanoparticles and hybrid materials containing metallic particles along with other (often carbon-based) constituent(s) at the nanometer level have been utilized for separation and pre-concentration of (ultra)trace elements in liquid samples. The studies included in this review emphasize the great analytical potential of procedures producing reliable results in the analysis of complex liquid matrices, where the detection of target analyte is often complicated by the presence of interfering substances.

## Introduction

**Figure 1:**
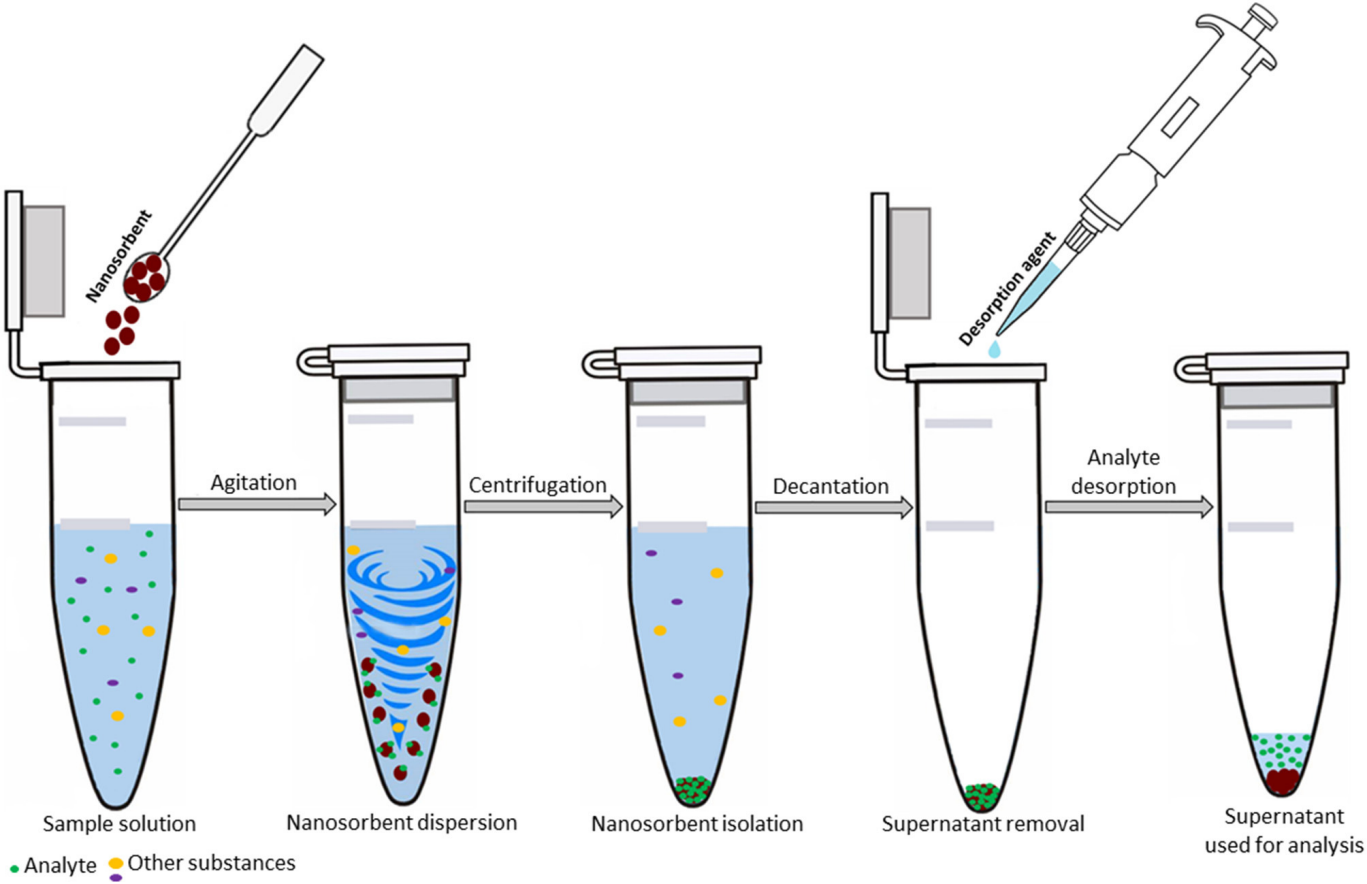
Illustration of the main steps of DMSPE procedure.

The separation techniques have rapidly gained popularity in recent years, as they represent a powerful tool to deal with the complexity of analyzed samples and (ultra)trace concentrations of studied analytes. Among separation techniques, the extraction procedures are particularly efficient in sample clean-up and analyte enrichment. Among these procedures, solid phase extraction (SPE), has become the most extensively used method in (ultra)trace analysis owing to many advantages over traditional separation techniques such as high extraction recoveries and high enrichment factors. In the last years, numerous modifications of this technique have been described in terms of different arrangements. Dispersive solid phase extraction (DSPE) as a novel alternative to conventional SPE is based on the dispersion of a solid sorbent in a liquid sample with no need for SPE column, extraction disc or cartridge. The advanced functional properties of the sorbent allow for direct interaction with the target analytes which favors the kinetics of the sorption and increases the efficiency of the extraction process. Another positive aspect related to this separation technique revolves around development and application of new and advanced sorption materials. The introduction of advanced sorbents favors the enhancement of selectivity toward the target analytes, higher sorption capacity and improvement of physicochemical and/or mechanical stability. The consumption of small amounts of sorbent (in the low milligram range) has led to a modification of the dispersive extraction, so-called dispersive micro-solid phase extraction (DMSPE). The DMSPE principle is similar to that of DSPE. After trapping the analyte on a sorbent dispersed in a liquid sample, the sorbent is usually isolated by centrifugation. The target analytes are eventually desorbed with a small amount of solvent appropriate for instrumental analysis. The DMSPE procedure is illustrated in Figure 1.

In DSPE procedures, reliable dispersion of the sorbent plays a crucial role. Dispersibility must be maintained during the whole extraction process, including the part where the analyte sorbs onto the sorbent and desorbs back into solution. For this reason, the dispersion needs to be assisted by the use of an external energy source (e.g., mechanical stirring) or chemicals. The different approaches allowing for the dispersion of a solid sorbent in DMSPE were compared by Chisvert et al. (2019).

The selection of a proper sorbent is an essential step toward development of reliable DMSPE procedure. While micro-sized materials of solid nature were commonly used at a time of early design stages of DMSPE, solid materials of a nanosize are preferably being used lately. Owing to their extremely small size and large surface area, they offer unique physical and chemical properties that are often very different from the properties of the same materials assembled at the micro- or macroscopic scale. In this regard, different nanoparticles of metallic nature have been tested as a new sorbent material for the separation and pre-concentration of organic and inorganic analytes in a number of environmental, biological, pharmaceutical, and food samples. The limits of detection (LODs) for particular analytes and the detection methods have been experimentally determined and are reported in Table 1.

**Table 1:** Summary table of the data obtained from experiments using metallic nanoparticles as sorbents in DMSPE procedures.

**Nanomaterial**	**m (mg)**	**Analyte**	**V (mL)**	**pH**	**Detection method**	**Desorption agent**	**LOD**	**RSD (%)**	**PF**	**References**
AgNPs	10	Hg	10	3.5	HR-CS ETAAS	7 M ${\text{HNO}}_{3}^{*}$	5.0 ng/L	6–11	15	Krawczyk and Stanisz, 2015
TiO_2_NPs	10	Hg	10	7.5	CVAAS	1 M ${\text{HNO}}_{3}^{**}$	4.0 ng/L	4–20	35	Krawczyk and Stanisz, 2016
ZnONPs	20	Ge	10	9.0	HR-CS ETAAS	H_2_O^**^	3.0 ng/L	4–23	15	Stanisz and Krawczyk-Coda, 2017
ZrNPs	20	Cd	30	7.0	SQT-FAAS	0.25 M HNO_3_	0.44 μg/L	1.9–6.3	116	Tekin et al., 2020
ZrNPs	20	Sb	15	2.0	SQT-FAAS	Conc. HCl	8.0 μg/L	3.4–4.6	180	Karlidag et al., 2020a
ZrNPs	10	Se	30	2.0	SQT-FAAS	Conc. HCl	5.3 μg/L	3.4–16.8	415	Karlidag et al., 2020b
ZrO_2_NPs	300	Ti	500	6.0	UV-Vis	11.3 M HF	0.10 μg/L	n/a	100	Zheng et al., 2009
Al_2_O_3_-Aliquat-336	100	Se	50	7.0	ICP-OES	1 M HNO_3_	1.4 ng/L	3.3	850	Nyaba et al., 2016
fibrous TiO_2_@g-C_3_N_4_	10	Sb	20	3.0	ICP-MS	1.5 M HNO_3_	0.37 ng/L	4.1	100	Chen et al., 2020a
fibrous TiO_2_@g-C_3_N_4_	15	Co	20	6.0	ICP-MS	0.5 M HNO_3_	0.12 ng/L	3.9	100	Chen et al., 2020b
fibrous TiO_2_@g-C_3_N_4_	15	Ni	20	6.0	ICP-MS	0.5 M HNO_3_	1.34 ng/L	4.8	100	Chen et al., 2020b
Al_2_O_3_/nano-G	1	Cr	25	6.5	EDXRF	No desorption	0.04 μg/L	3.5	n/a	Baranik et al., 2018a
Al_2_O_3_/GO	1	As	25	5.0	EDXRF	No desorption	0.02 μg/L	4.0	294	Baranik et al., 2018b
Al_2_O_3_/GO	1	Cr	25	6.0	EDXRF	No desorption	0.11 μg/L	4.0	953	Baranik et al., 2018b
MoS_2_-rGO	1	Cr	50	2.0	EDXRF	No desorption	0.05 μg/L	3.5	3350	Pytlakowska et al., 2020a
MoS_2_-rGO	1	Cr, Co, Ni, Cu, Zn, Pb	50	6.5	EDXRF	No desorption	0.07–0.12 μg/L	1.4–5.1	n/a	Pytlakowska et al., 2020b

*m, nanosorbent mass; V, sample volume; LOD, limit of detection; RSD, relative standard deviation; PF, pre-concentration factor; n/a, not available*;

* *sorbent containing the target analyte is dissolved and the concentration of the analyte in solution is determined*;

** *the analyte is captured on a solid support which is in the form of a slurry*.

Nanomaterials can be classified into different groups on the basis of their nature, composition, shapes, sizes, etc. In regard to their nature, nanomaterials are available in both natural and synthetic variations. In terms of their chemical composition, two main classes can be distinguished, inorganic (e.g., compounds containing metal or metal oxide) and organic (e.g., carbonaceous, polymeric materials). In recent years, a growing interest has been directed toward the use of hybrid organic-inorganic nanomaterials in which the functionality of organic compounds is combined with the stability of inorganic materials (Pyrzynska, 2020). The rising demand for hybrid multifunctional nanomaterials has stemmed from the need to enhance extraction efficiency. Whereas, mono-functional nanomaterials can offer only one type of interaction, the hybrid nanomaterials can improve extraction efficiency due to the synergistic effect resulting from interactions between different materials.

In terms of their structure, three main groups can be distinguished: nanoparticles (NPs), nanolayers (NLs), and nanotubes (NTs). Nanoparticles containing pure metals or their compounds in a core or shell are denoted as metallic. The class covers a variety of materials including metals (e.g., Au, Ag, Cu, Fe), metal oxides (e.g., TiO_2_, ZrO_2_, ZnO, CuO, Al_2_O_3_, Fe_x_O_y_), and other metal-containing NPs (e.g., CdSe/ZnS quantum dots). While there are also other materials used for nanolayers and nanotubes, carbon is still by far the best choice and therefore most research has been aimed toward the carbon ones. Metallic nanoparticles and carbonaceous nanomaterials have found their way into DMSPE procedures developed for efficient detection of various analytes, both organic and inorganic.

This minireview presents current DMSPE procedures for separation and/or pre-concentration of (ultra)trace elements which involve the use of metallic nanoparticles and hybrid materials consisting of metallic particles immobilized on surface of some advanced (mostly carbon-based) materials at the nano-scale.

## Metallic Nanoparticles and Hybrid Nanomaterials Containing Metallic Nanoparticles as SORBENTS for Efficient Trace Element separation and Pre-Concentration by DMSPE

Silver nanoparticles (AgNPs) and gold nanoparticles (AuNPs) have positioned themselves as one of the leading materials that are extensively used in various applications, including extraction procedures. Metal oxide nanoparticles, such as TiO_2_, ZrO_2_, ZnO, and Al_2_O_3_, belong to the family of amphoteric oxides and their application in extraction procedures allows for the extraction of both cations and anions (after the optimization of experimental conditions). Nanosized metal oxides act as ion-exchangers with large specific surface area and mesoporous structure, and are perceived as promising candidates for the sorption of various elements in aqueous media. Their thermal stability, good mechanical performance, and radiation resistance are definitely among the most significant benefits. However, particle size reduction is accompanied by changes in surface energy and affects the stability of the powder. With size reduced from micrometer to nanometer level, metal oxides have a tendency to form agglomerates due to interparticle forces, such as Van der Waals and electrostatic forces. Therefore, in nanoform they are relatively rarely used individually as adsorbents in extraction experiments (Karadjova et al., 2016). Combination of nanooxides with a carbonaceous support can eliminate this complication and give rise to a hybrid material with superior properties. It has been recognized that graphitization heat treatment of carbon fibers opens up new possibilities for the preparation of novel materials with increasing relevance in commercial as well as small-scale laboratory, industrial and research applications.

Carbon-based nanomaterials with a set of unique properties include graphite, diamond, glassy carbon, graphene, amorphous powders, carbon fibrous materials, carbon nanofibers (CNFs), and carbon nanotubes (CNTs) (Navrotskaya et al., 2020). This section is intended as a brief review of some representative studies on metallic nanoparticles combined with carbon-based nanostructures for the effective separation and pre-concentration of trace elements in aqueous media. By performing DSMPE measurements on presented types of nanomaterials, reliable results were achieved.

### Noble Metal Nanoparticles

Metallic nanoparticles containing valuable metals such as silver and gold exhibit unique optical, physical, chemical, and biological properties that are exploited in a wide range of applications (Hagarová, 2020). Their strong affinity for inorganic and organic mercury species can be utilized in extraction procedures for mercury binding. However, metallic nanoparticles containing noble metals can also be applied for the separation of other heavy metals (Li et al., 2020), organic species (pyrene, olefins, methylene blue) and gaseous species (NO, SO_2_) (Dastafkan et al., 2015).

Commercially available silver nanoparticles (AgNPs) have been lately used as solid sorbent in an ultrasound-assisted DMSPE (UA-DMSPE) procedure for separation and pre-concentration of Hg^2+^ ions in real spring water, tap water and ground water samples (Krawczyk and Stanisz, 2015). In this procedure, the adsorption of mercury on AgNPs is followed by separation of the liquid phase from the solid phase. Liquid phase is then removed and solid phase is immediately dissolved in 7 M HNO_3_. The mercury content is quantified by high-resolution continuum source electrothermal atomic absorption spectrometry (HR-CS ETAAS). The limit of detection (LOD) of this method has been calculated as 5 ng/L which demonstrates a great potential of the method in an ultratrace analysis of mercury ions in natural waters.

In a multistep process, Yordanova et al. (2014) prepared AgNPs supported on the surface of NH_2_-functionalized silica submicrospheres (SiO_2_/AgNPs) to be used as a sorbent for separation and pre-concentration of inorganic mercury in surface water samples during extraction procedure. This novel nanocomposite adsorbent was synthesized by a completely green procedure and was rigorously characterized by transmission electron microscopy (TEM), UV-Vis spectroscopy, X-ray diffraction (XRD), and atomic force microscopy (AFM).

Besides silver, utilization of gold in a form of nano-sized material in analytical chemistry has become a topic of interest during the last decades. The continued popularity of AuNPs can be attributed to their size- and shape- dependent optical and electrical properties and high electrocatalytic activity. Moreover, the surface chemistry of AuNPs is versatile, allowing the linking of various biofunctional groups (e.g., amphiphilic polymers, silanols, sugars, nucleic acids, proteins) through strong Au–S or Au–N bonding, or through physical adsorption (Lin et al., 2011). Such types of biomolecules can be adsorbed spontaneously onto gold surfaces generating well-organized, self-assembled monolayers (Khajeh et al., 2013), which favors these nanoparticles for application in procedures designed particularly for organic compounds separation.

Following the earlier work of Turkevich and Frens, Kiran (2015) synthesized AuNPs capped with 2-mercapto succinic acid in order to separate mercury from environmental water samples, vegetable, and crop samples collected near industrial areas. Nanoparticle structure and morphology was characterized by high-resolution transmission electron microscopy (HR-TEM) and scanning electron microscopy (SEM). The method was successfully applied to detect mercury up to ppb levels.

Palladium is considered the most valuable of the four major precious metals with extraordinary catalytic, powerful mechanical, and electroanalytical properties. Palladium nanoparticles (PdNPs) have been developed as self-therapeutics with proven anti-bacterial and cytotoxic pharmacological activity (Yaqoob et al., 2020). PdNPs have been applied as coprecipitation carriers for pre-concentration of Cu, Pb, and Cd in seawater and synthetic water samples prior to their measurement by atomic absorption spectrometry (AAS) (Zhuang et al., 1996). PdNPs have been found very useful in speciation studies conducted to determine Cr(VI) and Cr(III) in soil and aqueous media (Omole et al., 2007), and As(III) and As(V) in environmental water samples (Sounderajan et al., 2009).

The adsorption capacity of PdNPs as a DSPE sorbent for the separation and pre-concentration of selenium species in ground water has been evaluated by Kumar et al. (2015). The authors used sodium borohydrate for the reduction of Pd(II) to Pd(0) to obtain PdNPs. The same reducing agent was employed for the reduction of Se(IV) to Se(0). The reduced elemental selenium was then adsorbed on PdNPs surface. The PdNPs with adsorbed selenium were collected and dissolved in a minimum amount of nitric acid and the extracted selenium was quantified by electrothermal atomic absorption spectrometry (ETAAS). The adsorption capacity of PdNPs for Se(0) was 28 mg/g and the recoveries for Se(IV) and Se(VI) were in the range of 97–102%.

### Zirconium and Zirconium Dioxide Nanoparticles

Zirconium is a transition metal with extreme resistance to oxidation and corrosion which makes it ideal as an alloying agent choice for materials that are frequently exposed to corrosive environments (such as high temperatures, water, steam, acids, organic solvents, salts, etc.). Besides application of this metal in processes encountered in chemical engineering, zirconium and its compounds have found diverse uses in modern analytical chemistry as summarized by Pechishcheva et al. (2018). Very recently, the first work on application of zirconium nanoparticles (ZrNPs) in extraction procedures has been conducted.

Tekin et al. (2020) established a novel, green, highly accurate, and precise ZrNPs-based solid phase extraction strategy for separation and pre-concentration of trace cadmium prior to its quantification by slotted quartz tube-flame atomic absorption (SQT-FAAS). ZrNPs proved to be an effective adsorbent for the removal of Cd from water, the recovery rates of 96–100% were achieved.

ZrNPs have also been a choice of Karlidag and his co-workers who used it as sorbent material for the adsorption of antimony (Karlidag et al., 2020a) and selenium (Karlidag et al., 2020b) from tea samples by vortex assisted ligandless dispersive solid phase extraction (VA-LDSPE) and dispersive solid phase extraction (DSPE) method. Analyte quantification was performed by SQT-FAAS. Using Zr-NPs-VA-LDSPE-SQT-FAAS method, the analyte was detectable at concentrations 180-fold lower than those detected by conventional method. The obtained recovery values ranged between 93 and 102% for Sb and 92 and 101% for Se.

Zirconium dioxide (ZrO_2_), also known as zirconia, is a widely used inorganic material with exceptional biocompatibility, high mechanical and thermal stability, wear and corrosion resistance, chemical inertness and low systemic toxicity. However, in comparison with other metallic oxides used in extraction procedures for trace elements, it is considered less frequently used one. Yet, it has been successfully applied for the pre-concentration of Ti(IV) in water samples by Zheng et al. (2009) in a batch extraction procedure. Zirconium dioxide was in the form of ultrafine amorphous spherical powder prepared by precipitation in micro-droplets which act as nanoreactors in the microemulsion system composed of cyclohexane/water/TritonX-100/hexyl alcohol. The powder so obtained had particles substantially uniform in diameter and with low-aggregation behavior (Ma et al., 2004). The concentration of Ti(IV) was determined using a UV-1200 PC spectrophotometer. After optimization of experimental conditions, the authors achieved a limit of detection of 0.1 μg/L.

### Titanium Dioxide Nanoparticles

Titanium dioxide nanoparticles (TiO_2_NPs) have already shown outstanding performances in photoelectrochemical and photocatalytic processes. They have also found their application in extraction procedures as solid sorbents, owing to their optimal physicochemical properties (e.g., high thermal and chemical stability), high specific surface area, high adsorption capacity, low cost, and low toxicity (Krawczyk and Stanisz, 2016). TiO_2_NPs have been used for separation and pre-concentration of various trace elements in extraction procedures (Xu et al., 2018).

The method combining ultrasound-assisted DMSPE (UA-DMSPE) with cold vapor atomic absorption spectrometry (CVAAS), where TiO_2_NPs were successfully used for separation and pre-concentration of total mercury and mercury species (Hg^2+^ and CH_3_Hg^+^) in biological, geological, and water samples, has been presented by Krawczyk and Stanisz (2016). After extraction, a sorbent with entrapped analyte was separated from the bulk solution and mixed with 1 M HNO_3_ to prepare a slurry that was then measured for mercury. However, it is known that detection limits that can be achieved using CVAAS after pre-concentration on TiO_2_NPs are better than those obtained with conventional CVAAS.

In a study by Chen et al. (2020a), the fibrous TiO_2_@g-C_3_N_4_ nanocomposites (FTGCNCs) were tested as a new adsorbent for separation of various antimony species [Sb(III), Sb(V), residual, digestible, and total Sb] in a cow milk by DMSPE prior to inductively coupled plasma mass spectrometry (ICP-MS) analysis. The results showed that Sb(III) was quantitatively adsorbed on FTGCNCs in the pH range of 2–4, while Sb(V) remained in an aqueous phase. Compared to other separation and detection methods, particularly solid-phase microextraction coupled to inductively coupled plasma mass spectrometry (SPME-ICP-MS), ion chromatography coupled to inductively coupled plasma mass spectrometry (IC-ICP-MS), magnetic solid phase extraction coupled to inductively coupled plasma mass spectrometry (MSPE-ICP-MS), high-performance liquid chromatography coupled to inductively coupled plasma mass spectrometry (HPLC-ICP-MS), cloud point extraction combined with electrothermal vaporization inductively coupled plasma mass spectrometry (CPE-ETV-ICP-MS) and hydride generation inductively coupled plasma mass spectrometry (HG-ICP-MS), this approach allows for more accurate determination of sample composition and results in a lower LOD [0.37 pg/mL for Sb(III)] and higher enrichment factor (100).

The efficiency of FTGCNCs as sorbent material was supported in their following study (Chen et al., 2020b), where FTGCNCs were used for DMSPE of Co and Ni prior to their determination by ICP-MS. The apparent selectivity of this material for some metal ions can be attributed to a large number of –NH_2_, –NH– and =N functional groups distributed on FTGCNCs' surface. For Co and Ni retention on FTGCNCs, the optimal pH ranges between 5 and 8.

Although graphitic carbon nitride (g-C_3_N_4_) shows great potential as a new sorbent in future sample pretreatment techniques, it has some issues from the DMSPE point of view. Pure g-C_3_N_4_ easily aggregates which results in a decrease of its specific surface area (Sun et al., 2016). The weak polarity of g-C_3_N_4_ causes its poor dispersion in water. Fortunately, these problems can be solved by the *in-situ* growth of g-C_3_N_4_ on the surface of TiO_2_ nanofibers (TDNFs) to form the fibrous TiO_2_@g-C_3_N_4_ nanocomposites (FTGCNCs).

### Zinc Oxide Nanoparticles

The features such as large specific surface area, high porosity, low toxicity, and easy preparation have ensured great success of zinc oxide nanoparticles (ZnONPs) throughout the field of chemistry. In SPE procedures, ZnONPs have found application as effective nanosorbents. This material has been successfully used for efficient separation and pre-concentration of trace germanium in various food samples including tea leaves and mixed herbs by an ultrasound-assisted dispersive micro-solid phase extraction (UA-DMSPE) prior to its determination by high-resolution continuum source electrothermal atomic absorption spectrometry (HR-CS ETAAS) (Stanisz and Krawczyk-Coda, 2017).

Zinc oxide is a unique material with versatile industrial and commercial applications. However, there is a constant race toward the performance enhancement. Doping is one of the methods commonly used for nanoparticle modification in order to enhance their electrical, optical, physical, biological, and chemical properties. Doping can increase the surface area, reduce the mass, and alter nanoparticle morphology resulting in optimized functional properties of nanomaterials (Khan et al., 2016). Although the methodology of the following work is based on thermodynamic and kinetic principles, the general steps followed in dispersive extraction (e.g., addition of 25 mg of Ca-doped ZnONPs to the metal ion solution) classify this procedure as DMSPE. Khan et al. (2016) developed simple, sensitive and innovative Ca-doped ZnONPs extraction system for the recognition and elimination of Pb(II) ions in real water samples (drinking water, tap water, lake water, and seawater) based on a high selectivity of Ca-doped ZnONPs for Pb(II). Although this sorbent material was also applied for the extraction of Cd(II), Cu(II), Mn(II), Hg(II), Pd(II), La(III), and Y(III), it was found to be highly selective for Pb(II). For this metal ion, the extraction efficiency of 94–99% was achieved. The adsorption process for Pb(II) was characterized as monolayer adsorption with the adsorption capacity of 84.66 mg/g. Nanoparticle structure was studied by field emission scanning electron microscope (FESEM), X-ray microanalysis (EDS), X-ray diffraction (XRD), X-ray Photoelectron (XPS), Fourier transform infrared spectroscopy (FTIR), and UV-Vis spectrophotometry.

### Aluminum Oxide Nanoparticles

Aluminum oxide has become a typical sorbent material in SPE procedures. The high-performance of Al_2_O_3_ nanoparticles (Al_2_O_3_NPs) is attributed to a large specific surface area, high adsorption capacity, mechanical strength, high chemical activity, enhanced sorption kinetics, and low-temperature modification (Mohammadifar et al., 2015).

Hassanpoor et al. (2015) proposed a simple and efficient method for the extraction and speciation of trace quantities of arsenic in spiked real water, food and biological samples. For this purpose, Al_2_O_3_NPs were functionalized by a ligand containing donor atoms such as oxygen, nitrogen, and sulfur [3,3′-bis-(3-triethoxysilylpropyl)-2,2′-dithioxo [5,5′] bithiazolidinylidene-4,4′-dione]. Desorption of the analyte was done by 1 M HCl and final measurements were performed by ETAAS. The adsorption capacity of the sorbent was tested in multiple cycles of adsorption and desorption. The obtained results showed that functionalized Al_2_O_3_NPs could be reused up to 70 times without any considerable loss in adsorption efficiency.

The different modification of Al_2_O_3_NPs used for selenium speciation in surface water samples by DMSPE procedure has been presented in a work published by Nyaba et al. (2016). In this study, Al_2_O_3_NPs were functionalized with quaternary ammonium salt (Aliquat336) and characterized by FTIR. Desorption of the analyte was done by diluted HNO_3_ and its quantification was performed by inductively coupled optical emission spectrometry (ICP-OES). The introduction of DMSPE prior to ICP-OES detection resulted in a significantly low LOD and LOQ (limit of quantification). The optimum pH for successful separation of inorganic Se species in sample solution was between 2 and 7. The Se(IV) recovery was >90%, however, the recovery of Se(VI) was only near 5% over most of the pH range.

Baranik et al. (2018a,b) obtained novel aluminum oxide/graphene oxide and aluminum oxide/nano-graphite sorbents for As(V), Cr(III), and Cr(VI) pre-concentration in natural waters by means of (ultrasound-assisted-) DMSPE. Graphene oxide (GO) was synthesized by improved Hummers' method (Marcano et al., 2010) out of high purity graphite, sodium nitrate, sulphuric acid, potassium permanganate, and hydrogen peroxide. Alumina supported on graphene oxide (Al_2_O_3_/GO) nanocomposite was prepared by a simple sono-thermo-chemical method involving dispersion of GO in a high purity water followed by addition of Al(NO_3_)_3_·9H_2_O. After an ultrasonic bath treatment followed by drying at 100°C, the solid was heated at 500°C in a furnace to initiate the growth of Al_2_O_3_NPs on the GO surface. The aluminum oxide coated nano-graphite (Al_2_O_3_/nano-G) was synthesized out of Al(NO_3_)_3_·9H_2_O, Triton X-100 and high purity graphene nanosheets in a sono-thermo-chemical process. Thus, prepared adsorbents were characterized by SEM, TEM, powder XRD, and Raman spectroscopy.

**Figure 2:**
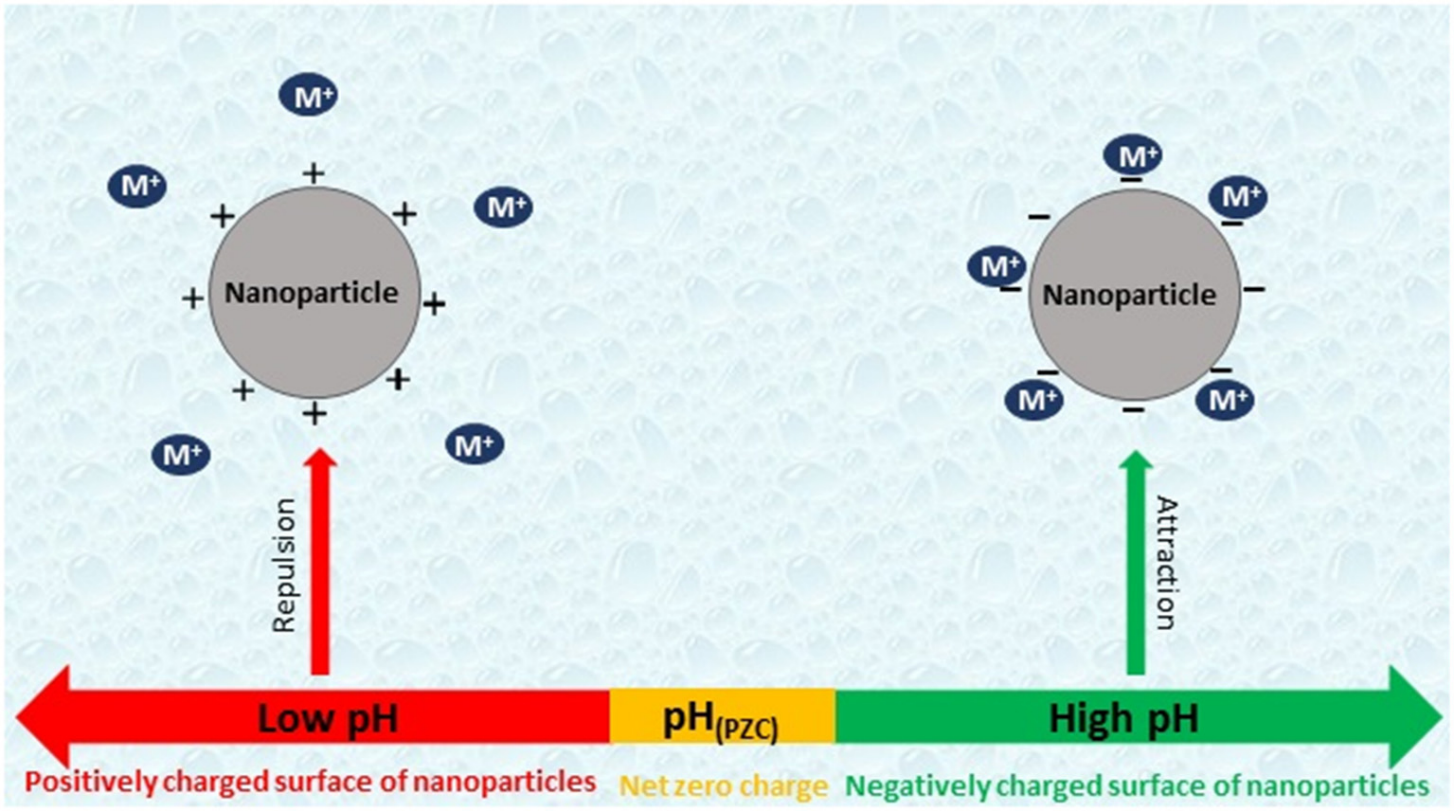
The interaction between positively- and negatively-charged surface of a nanoparticle and positively charged metal ions (M^+^) in a solution at pH values below and above the point of zero charge (PZC).

Al_2_O_3_/nano-G has exhibited high selectivity for Cr(III) in the presence of Cr(VI). Al_2_O_3_/GO has demonstrated selectivity toward arsenates in the presence of arsenites at pH 5 and chromium(III) ions in the presence of chromate anions at pH 6. Under optimized conditions, As(V) and Cr(III) ions could be determined with very good precision (RSD 2.7–4%), excellent LOD (0.02 ng/mL As and 0.11 ng/mL Cr) and recovery rates (92–108%). Compared to Al_2_O_3_/GO nanocomposite, Al_2_O_3_/nano-G has been characterized by better LOD values for Cr(III). Figure 2 illustrates the attraction/repulsion between positively charged metal ions and positively- and negatively-charged surface of a nanoparticle in a different pH environment.

### Molybdenum Disulfide Nanoparticles

Pytlakowska et al. (2020a,b) ran experiments on two variants of graphene, graphene oxide (GO) and reduced graphene oxide (rGO), coupled with molybdenum disulfide (MoS_2_) in order to investigate the potential of the hybrid material for the adsorption of metal ions. MoS_2_ was supported on GO by hydrothermal method. The resulting nanocomposite (MoS_2_-rGO) was characterized by XPS, SEM and TEM. The material was successfully applied for chromium speciation in real lake, spring and river water samples and artificial sea water by UA-DMSPE method. At pH 2, MoS_2_-rGO showed a strong affinity for Cr(VI) leaving Cr(III) ions in an aqueous solution. The strong affinity of a target analyte toward MoS_2_-rGO NPs results from both electrostatic interaction and outsphere surface complexation. The adsorption capacity of 583.5 mg/g was nearly 6.5 times higher than that reported for untreated MoS_2_. The adsorption process was predominantly ruled by chemisorption.

Reduced graphene oxide (rGO) decorated with molybdenum disulfide (MoS_2_) was synthesized by hydrothermal method and studied by different spectroscopic techniques, such as FTIR, Raman spectroscopy and XPS. The detailed high resolution images of the hybrid material were provided by SEM and TEM. MoS_2_-rGO has been evaluated as a solid nanoadsorbent for the pre-concentration of heavy metal ions in different water samples by DMSPE prior to energy dispersive X-ray fluorescence spectrometry (EDXRF) analysis. The maximum adsorption capacities were ~1.5–5-folds higher than those of unmodified GO; *q*_*max*_were 235.1, 130.8, 279.3, 414.8, 483.7, 381.9 mg/g for Cr(III), Co(II), Ni(II), Cu(II), Zn(II), and Pb(II) ions, respectively. The introduction of ultrasound in adsorption process has resulted in excellent recovery rates (close to 100% after 10 min of sonication).

## Summary

Metallic nanoparticles (MNPs) have found varying applications on an industrial scale, this boom has also affected the area of analytical chemistry. MNPs are now heavily utilized in many extraction procedures for the separation/pre-concentration of organic (Sajid et al., 2021; Wu et al., 2021) and inorganic (Filik and Avan, 2019; Er et al., 2020) analytes from environmental, biological, pharmaceutical, and food samples. However, the application of MNPs in column experiments is rather rare, probably due to their tendency to form agglomerates (Karadjova et al., 2016; Hashemi et al., 2018). In contrast, dispersive extraction techniques utilizing MNPs are reported more frequently, whether it is analytical research aimed at reliable quantification of different analytes present in aqueous samples (often at ultratrace concentration levels) or comparative studies on the removal of toxic pollutants from contaminated liquid matrices. The latter mainly involve thermodynamic and kinetic studies on the assessment of sorption capacity of the sorbents.

Our minireview summarizes the current knowledge on the latest trends in dispersive extraction, the so-called dispersive micro-solid phase extraction, where only a few units or tens of milligrams of nanosorbent are required. The focus is mainly on metal nanoparticles utilized for the separation and pre-concentration of (ultra)trace inorganic analytes in DMSPE procedures. The examples of papers that have been published in recent years highlight the strong analytical potential of procedures that employ MNPs (either commercially available or synthesized in-house) and hybrid nanomaterials containing MNPs coupled with another (often carbon-based) nanomaterial.

Analyzing the data in Table 1, the following findings can be highlighted. Besides the desorption of the analyte carried out with an appropriate desorbing agent, which is the most common procedure performed in the last step of DMSPE before analyte quantification, other options had also been discussed. The first option involves dissolution of the nanosorbent material carrying adsorbed analyte and using the resulting solution for analysis (Krawczyk and Stanisz, 2015). Alternatively, a fine suspension of the sorbent containing adsorbed analyte may be prepared in the selected reagent and thus obtained “slurry” is then analyzed (Krawczyk and Stanisz, 2016). There are also studies in which no desorption procedure has been outlined. Instead, following extraction, the sorbent and the adsorbed analyte have been filtered off, dried and submitted for analysis along with the filter (Baranik et al., 2018a,b; Pytlakowska et al., 2020a,b). This type of procedure appears to be advantageous if the analyte is quantified by means of energy dispersive X-ray fluorescence spectrometry (EDXRF). The enrichment factor (EF) of 3350, which was calculated as a ratio of sensitivity of DMSPE–EDXRF procedure to sensitivity of the direct EDXRF measurement and reported by Pytlakowska et al. (2020a), can be considered abnormally high.

Similarly to other extraction techniques developed for pre-concentration of the analyte of interest, the DMSPE approach allows for a several-fold reduction of the limits of detection (LODs). The lowest LODs were achieved with DMSPE-ICP-MS (Chen et al., 2020a,b). However, the LOD of 1.4 ng/L obtained when combining DMSPE and ICP-OES (at EF of 850) indicates that after optimization of the experimental conditions even a quantification method associated with significantly higher LODs can be reliably applied in ultratrace analysis. (In general, the LODs for ICP-OES based methods are very high compared to those obtained by ICP-MS and ETAAS. The LOD obtained by this method are broadly comparable with those reported for FAAS and application of this method for the direct determination of ultratrace concentrations of particular elements is rather rare.) The integration of DMSPE with HR-CS-ETAAS or CVAAS leads to further improvement with respect to LODs, the levels as low as ng/L indicate that there is a great potential for the use of such arrangements in ultratrace analysis of inorganic analytes (Krawczyk and Stanisz, 2015, 2016; Stanisz and Krawczyk-Coda, 2017). The precision of the developed procedures [evaluated with relative standard deviation (RSD) values] has been found acceptable in all studies, although some works have reported RSD above 10%. In a recent study on UA-DMSPE extraction method coupled with CVAAS conducted by Krawczyk and Stanisz (2016), high RSDs (6–20%) were observed for methylmercury in real water samples and biological material (Dogfish liver), which could be related to indirect detection approach for this analyte. RSDs for total Hg and Hg^2+^ measurements ranged from 4 to 11%. Performing quantification of Ge in real plant-based food samples by UA-DMSPE-HR-CS-ETAAS, the highest RSD (23%) was reported for white tea samples, where the analyte concentration was close to the detection limit (Stanisz and Krawczyk-Coda, 2017). In instances where the analyte concentration is below the quantification limit (LOQ), even RSD values around 20% are considered acceptable and the numerical value obtained for analyte concentration in the sample should be taken with some caution. The extraction yields reported in “recovery studies” indicate that monitored analytes were extracted quantitatively from different types of real samples.

If we realize that the amount of sorbent as low as 1 mg added in tens of mL (25 and 50 mL) of sample is still sufficient to deliver reliable separation and pre-concentration of the target analyte even in the presence of a wide spectrum of possible interferents, it is hard to argue that the capabilities and achievements of these techniques have been anything other than remarkable.

## Author Contributions

IH and LN prepared the manuscript, made its revision, and final corrections. Both authors contributed to the article and approved the submitted version.

## Conflict of Interest

The authors declare that the research was conducted in the absence of any commercial or financial relationships that could be construed as a potential conflict of interest.
